# Association of Surrogate Decision-making Interventions for Critically Ill Adults With Patient, Family, and Resource Use Outcomes: A Systematic Review and Meta-analysis

**DOI:** 10.1001/jamanetworkopen.2019.7229

**Published:** 2019-07-19

**Authors:** Lior Bibas, Maude Peretz-Larochelle, Neill K. Adhikari, Michael J. Goldfarb, Adriana Luk, Marina Englesakis, Michael E. Detsky, Patrick R. Lawler

**Affiliations:** 1 Interdepartmental Division of Critical Care, University of Toronto, Toronto, Ontario, Canada; 2 Sunnybrook Research Institute, Department of Critical Care Medicine, Sunnybrook Health Sciences Centre, Toronto, Ontario, Canada; 3 Division of Cardiology, Department of Medicine, McGill University, Montreal, Quebec, Canada; 4 Peter Munk Cardiac Centre, University Health Network, Toronto, Ontario, Canada; 5 Library and Information Services, University Health Network, Toronto, Ontario, Canada; 6 Sinai Health System, Toronto, Ontario, Canada; 7 Heart and Stroke/Richard Lewar Centre of Excellence, University of Toronto, Toronto, Ontario, Canada

## Abstract

**Importance:**

Physicians often rely on surrogate decision-makers (SDMs) to make important decisions on behalf of critically ill patients during times of incapacity. It is uncertain whether targeted interventions to improve surrogate decision-making in the intensive care unit (ICU) reduce nonbeneficial treatment and improve SDM comprehension, satisfaction, and psychological morbidity.

**Objective:**

To perform a systematic review and meta-analysis of randomized clinical trials (RCTs) to determine the association of such interventions with patient- and family-centered outcomes and resource use.

**Data Sources:**

A search was conducted of MEDLINE, Embase, and other relevant databases for potentially relevant studies from inception through May 30, 2018.

**Study Selection:**

Randomized clinical trials studying interventions that were targeted at SDMs or family members of critically ill adults in the ICU were included. Key search terms included *surrogate* or *substitute decision-maker*, *critically ill*, *randomized controlled trials*, and their respective related terms.

**Data Extraction and Synthesis:**

This study followed the Preferred Reporting Items for Systematic Reviews and Meta-analyses (PRISMA) guidelines. Two independent, blinded reviewers independently screened citations and extracted data. Random effects models with inverse variance weighting were used to pool outcomes data when possible and otherwise present findings qualitatively.

**Main Outcomes and Measures:**

Outcomes of interest were divided into 3 categories: (1) patient-related clinical outcomes (mortality, length of stay [LOS], duration of life-sustaining therapies), (2) SDM and family-related outcomes (comprehension, major change in goals of care, incident psychological comorbidities [posttraumatic stress disorder, anxiety, depression], and satisfaction with care), and (3) use of resources (cost of care and health care resource use).

**Results:**

Of 3735 studies screened, 13 RCTs were included, comprising a total of 10 453 patients. Interventions were categorized as health care professional led (n = 6), ethics consultation (n = 3), palliative care consultation (n = 2), and media (n = 1 pamphlet and 1 video). No association with mortality was observed (risk ratio, 1.03; 95% CI, 0.98-1.08; *P* = .22). Intensive care unit LOS was significantly shorter among patients who died (mean difference, −2.11 days; 95% CI, −4.16 to −0.07; *P* = .04), but not in the overall population (mean difference, –0.79 days; 95% CI, −2.33 to 0.76 days; *P* = .32). There was no consistent difference in SDM-related outcomes, including satisfaction with care or perceived quality of care (n = 6 studies) and incident psychological comorbidities (depression: ratio of means, −0.11; 95% CI, −0.29 to 0.08; *P* = .26; anxiety: ratio of means, −0.08; 95% CI, −0.25 to 0.08; *P* = .31; or posttraumatic stress disorder: ratio of means: −0.04; 95% CI, −0.21 to 0.13; *P* = .65). Among 6 trials reporting effects on health care resource use, only 1 nurse-led intervention observed a significant reduction in costs ($75 850 control vs $51 060 intervention; *P* = .04).

**Conclusions and Relevance:**

Systematic interventions aimed at improving surrogate decision-making for critically ill adults may reduce ICU LOS among patients who die in the ICU, without influencing overall mortality. Better understanding of the complex processes related to surrogate decision-making is needed.

## Key Points

**Question:**

Are intensive care unit-based interventions aimed at surrogate decision-makers associated with differences in patient-centered outcomes and improved surrogate decision-maker–centered outcomes?

**Findings:**

In this systematic review and meta-analysis of 13 randomized clinical trials including 10 453 patients, diverse interventions were associated with a 2-day reduction in intensive care unit length of stay only among patients who died, without an association with overall mortality. Associations between these interventions and surrogate decision-maker–related outcomes were inconsistent.

**Meaning:**

Intensive care unit-based interventions aimed at improving surrogate decision-making may lead to shorter intensive care unit stays in dying critically ill patients.

## Teaser

This systematic review and meta-analysis examines the association of interventions used to improve decision-making by surrogates of patients in intensive care units with patient- and family-centered outcomes and resource use.

## Introduction

Patients with critical illness are often unable to convey their values and preferences and make autonomous decisions regarding their care.^1^ In such situations, physicians rely on patients’ surrogate (or substitute) decision-makers (SDMs), often family members, to make clinical decisions regarding the patient’s care using a shared-decision-making model.^2^ However, shared decision-making between clinicians and SDMs can be challenging, leading to potential conflicts and diminished satisfaction with care.^3,4^ Furthermore, SDMs may correctly predict patients’ treatment preferences in only about two-thirds of the cases.^5^ The burden of assuming such a critical yet challenging role for loved ones can lead to long-lasting negative sequelae, including posttraumatic stress disorder (PTSD), anxiety, and depression.^6,7,8^

Surrogate decision-making and family-centered interventions have been proposed to improve shared decision-making and alignment with patients’ values and SDM and family’s needs.^9,10^ Examples of such family-centered interventions include structured approaches to physician-family communication, formal training of health care professionals, proactive palliative care and ethics consultations, or the use of information leaflets.^10^

Despite their potential value, it is unclear whether such interventions can be meaningfully and systematically deployed in intensive care units (ICUs) and whether they are associated with improved patient- and SDM-related outcomes. We therefore performed a systematic review and meta-analysis of randomized clinical trials (RCTs) assessing the reported effects of communicational and educational interventions targeting SDMs in the ICU and examined their association with patient-related and SDM-related outcomes, as well as use of resources.

## Methods

### Search Strategy

An experienced information specialist (M.E.) searched MEDLINE (OVID interface), MEDLINE In-Process/ePubs ahead of print, Embase, Ovid Nursing Database, Cochrane Central Register of Controlled Trials, and Cochrane Database of Systematic Reviews via OVID interface and PubMed (non-MEDLINE records only) for potentially relevant studies (from database inception to May 30, 2018). Controlled vocabulary terms and text words relating to substitute decision-makers and RCTs or systematic reviews or meta-analyses were used, such as *surrogate* or *substitute decision-maker*, *critically ill*, *randomized controlled trials*, and their respective related terms. Results were limited to English- or French-language publications. The full search strategy is provided in eTable 1 in the Supplement. We also screened reference lists of included trials and relevant narrative reviews. This study followed the Preferred Reporting Items for Systematic Reviews and Meta-analyses (PRISMA) reporting guideline and was registered on PROSPERO (CRD42018077654) prior to data extraction.^11^

### Study Selection

We included RCTs performed in ICUs involving interventions that were targeted, fully or in part, at SDMs or family members. We excluded trials with exclusively pediatric (age <18 years) patients, interventions aimed only at health care professionals, and those in which interventions were entirely performed outside of the ICU. We also excluded conference abstracts.

Outcomes of interest were divided into 3 general categories: (1) patient-related clinical outcomes (ICU and hospital mortality, ICU and hospital length of stay [LOS], duration of life-sustaining therapies [mechanical ventilation, vasopressors, nutritional support], and self-reported satisfaction with care, psychological symptoms, and functional status); (2) SDM and family-related outcomes (comprehension, change in goals of care status as decided by the SDM, development of psychological comorbidities [PTSD, anxiety, depression], and self-reported satisfaction with care); and (3) use of resources (cost of care and health care resource use).

### Data Extraction and Assessment of Studies

Two independent blinded reviewers (L.B., M.P.-L.) independently screened citations for potentially relevant studies using the web application Rayyan,^12^ and the full texts of studies deemed potentially relevant by either reviewer were reviewed in duplicate. For trials meeting inclusion criteria, we extracted study-level data, including author, year of publication, study design, setting, population, intervention, control, and outcomes. Authors of studies reporting mortality and ICU LOS were contacted to clarify data (eg, to provide an SD for a reported mean value). We used the Cochrane Collaboration tool for assessing risk of bias to further examine the quality of each study.^13^ Both reviewers attributed a score of low, unclear, or high for each of the following bias categories: random sequence generation, allocation concealment, blinding of participants, personnel and outcome assessment, incomplete outcome reporting, and selective outcome reporting.

### Statistical Analysis

We used random-effects models with inverse variance weighting to pool outcomes data from each trial. Pooled data are reported as relative risk (RR) for mortality, weighted mean difference for ICU LOS, and ratio of means^14^ (experimental group/control group) for psychological comorbidities (ie, depression, anxiety, PTSD). All estimates are reported with 95% CIs. For ICU LOS, we examined pooled outcomes data in all patients as well as in those who died in the ICU. Data from cluster RCTs were adjusted for design effect using the intracluster correlation coefficient.^13,15^ For the studies that reported ICU LOS as medians, we contacted the primary author to obtain the corresponding mean and SD. For studies in which the authors did not provide additional data, we estimated the mean and SD using validated estimations based on the median and interquartile range.^16^ We considered 2-sided *P* ≤ .05 as statistically significant. For each outcome, we estimated between-study statistical heterogeneity using *I*^2^, which is the percentage of total variability across studies attributable to true heterogeneity rather than chance, and we interpreted the *I*^2^ value as low (25%-49%), moderate (50%-74%), or high (≥75%).^17,18^ Outcomes of interest not amenable to meta-analysis due to heterogeneity of definitions or reporting are presented for each trial separately. Funnel plots and assessment of publication bias using the Peters test^19^ were performed for meta-analyzed outcomes derived from 10 or more studies.^20^ Data analysis was performed using RevMan, version 5.3 statistical software (The Cochrane Collaboration).

## Results

### Search Results and Study Characteristics

**Figure 1.**
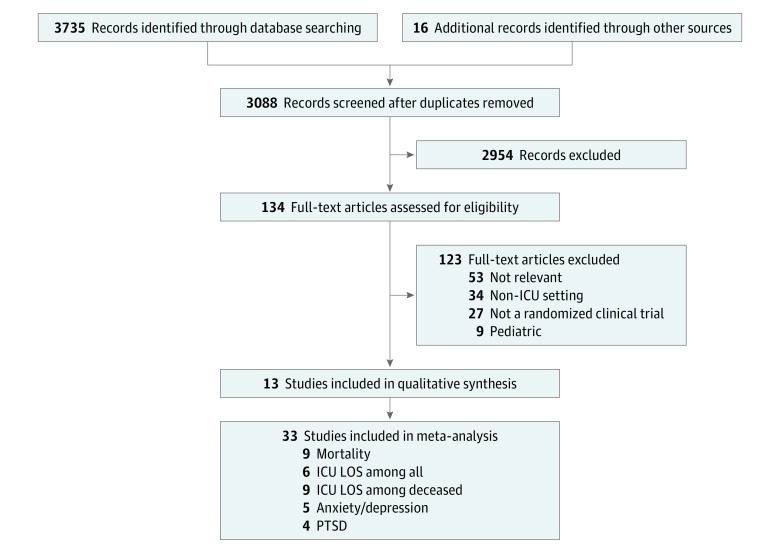
Flow Diagram ICU indicates intensive care unit; LOS, length of stay; and PTSD, posttraumatic stress disorder.

Of 3735 records identified, 3088 were screened after duplicates were removed (Figure 1), 11 RCTs met inclusion criteria and another 2 were found after screening the references of relevant records, for a total of 13 RCTs, comprising a total of 10 453 patients. The number of corresponding family members and SDMs was inconsistently reported.

Trial characteristics are summarized in the Table. Two trials were cluster RCTs,^21,22^ 1 trial was a step-wedge cluster RCT,^26^ and 10 trials were individually randomized.^23,24,25,27,28,29,30,31,32,33^ Five trials were single centered,^25,27,28,31,33^ whereas 8 were multicentered.^21,22,23,24,26,29,30,32^ Ten studies were set in the United States,^21,22,23,25,26,27,28,29,30,33^ 2 were set in France,^24,32^ and 1 was conducted in Australia.^31^ All trials were unblinded to participants, family members, physicians, and researchers. Risk of bias assessment is provided in eTable 2 in the Supplement.

**Table.** Characteristics of Included Trials

Category	Source	Design	Sites	Setting	Intervention	Population	No.	
							Patients	Family Members/SDMs
Health care professional	Connors et al,^21^ 1995	Cluster RCT	Multicenter	5 Academic hospitals; United States	Improved communication with family, including nurse acting as facilitator	Patients with advanced stages of various life-threatening illnesses	4804	NR
	Curtis et al,^22^ 2011	Cluster RCT	Multicenter	12 Hospitals (4 teaching, 8 non-teaching); United States	Quality improvement and educational intervention targeted at physicians	Patients dying in the ICU or within 30 h of ICU discharge	2318	822
	Curtis et al,^23^ 2016	RCT	Multicenter	5 ICUs in 2 hospitals (1 academic, 1 community); United States	Communication with trained facilitator (nurse or social worker)	Patients receiving MV with ≥30% risk of dying	168	268
	Lautrette et al,^24^ 2007	RCT	Multicenter	22 ICUs; France	Improved end-of-life conference and bereavement leaflet	Patients who would die within a few days, as decided by physician	126	126
	Torke et al,^25^ 2016	RCT	Single center	ICU in tertiary care hospital; United States	Dedicated trained nurse acting as Family Navigator	Sedated or comatose ICU patients	26	26
	White et al,^26^ 2018	Step-wedged, cluster RCT	Multicenter	5 ICUs (4 academic, 1 community); United States	Multicomponent, family-support intervention delivered by the interprofessional ICU team	Patients with ≥1 of MV for ≥4 days or >40% chance of death during hospitalization or >40% chance of severe functional impairment	1420	1106
Ethics	Andereck et al,^27^ 2014	RCT	Single center	Tertiary care ICU; California	Proactive ethics intervention	Patients with ICU LOS ≥5 d	384	319
	Schneiderman et al,^28^ 2000	RCT	Single center	Tertiary care ICU; California	Ethics consultation offered	Patients in whom treatment conflicts were identified	70	NR
	Schneiderman et al,^29^ 2003	RCT	Multicenter	7 Hospitals (5 academic, 2 community); United States	Ethics consultation offered	Patients in whom treatment conflicts were identified	551	525
Palliative care	Carson et al,^30^ 2016	RCT	Multicenter	4 Hospitals (3 tertiary care, 1 community); United States	Multiple family meetings led by palliative care team and brochure	Patients receiving MV ≥7 d	256	365
	Cheung et al,^31^ 2010	RCT	Single center	Tertiary care ICU; Australia	Consultation from palliative care team	Patients in whom the treating intensivist believed treatment should not be escalated	20	9
Media	Azoulay et al,^32^ 2002	RCT	Multicenter	34 ICUs; France	Family information leaflet	Expected ICU LOS >48 h	175	175
	Wilson et al,^33^ 2015	RCT	Single center	Tertiary care ICU; United States	8-min Education video	All patients admitted to ICU	135	65

Abbreviations: ICU, intensive care unit; LOS, length of stay; MV, mechanical ventilation; NR, not reported; RCT, randomized clinical trial; SDMs, surrogate decision-makers.

All studies required critically ill patients to have an available SDM. The interventions varied between trials, although all aimed to improve SDM communication and understanding: 6 involved health care professional–led interventions (4 nurse led and 2 physician led),^21,22,23,24,25,26^ 3 consisted of an ethics consultation,^27,28,29^ 2 involved palliative care teams,^30,31^ and 2 used media tools (1 pamphlet and 1 video).^32,33^ All control groups consisted of usual care provided by the centers’ ICU. Twelve trials were conducted exclusively within the ICU. The SUPPORT study also included ICU and non-ICU hospitalized patients with advanced stages of severe life-threatening illnesses (median APACHE score, 32).^21^ Nine trials included patients in the ICU who were at higher risk of dying, as decided by the physician or by an increased LOS or length of mechanical ventilation.^21,22,23,24,26,27,30,31,32^ Two trials evaluating a proactive ethics consultation only included patients and SDMs in whom treatment conflicts were identified regardless of patient prognosis,^28,29^ 1 trial included all patients admitted to the ICU,^33^ and another included all sedated or comatose patients in the ICU.^25^ Individual patient-centered, family-centered, and resource use outcomes are summarized in eTable 3 in the Supplement.

### Patient-Related Clinical Outcomes

**Figure 2.**
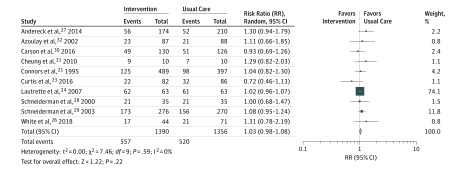
All-Cause Mortality The size of the box represents the statistical weight of each trial.

Nine of the 10 RCTs reporting mortality did not observe mortality differences.^21,23,24,26,27,28,29,30,31,32^ Although an initial increase in in-hospital mortality was seen with multicomponent intervention by White et al,^26^ this difference dissipated by 6 months. Meta-analysis of these 10 trials demonstrated no overall association with mortality in aggregate (RR, 1.03; 95% CI, 0.98-1.08; *I*^2^ = 0%) (Figure 2).

**Figure 3.**
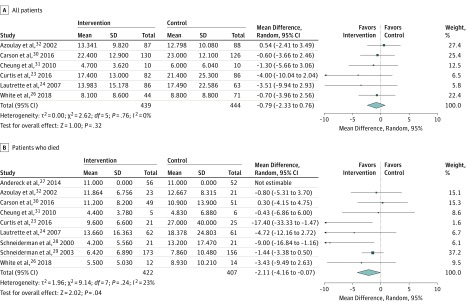
Length of Stay in Days in the Intensive Care Unit The size of the box represents the statistical weight of each trial.

In the 6 RCTs reporting ICU LOS among all patients,^23,24,26,30,31,32^ interventions were not associated with a significant difference in LOS (mean difference, −0.79 days; 95% CI, −2.33 to 0.76 days; *P* = .04; *I*^2^ = 0%) (Figure 3A). However, when restricted to patients who did not survive (n = 9 trials),^23,24,26,27,28,29,30,31,32^ meta-analysis demonstrated a significant reduction in ICU LOS (mean difference, −2.11 days; 95% CI, −4.16 to −0.07 days; *P* = .04; *I*^2^ = 23%) (Figure 3B). Statistical heterogeneity, as measured by the *I*^2^ measure, was estimated to be low for overall mortality, ICU LOS in all patients, and ICU LOS among patients who died. There was no evidence of publication bias for the 10 studies included for mortality, using the Peters test. The funnel plot for mortality can be found in eFigure 2 in the Supplement.

Nine RCTs assessed end-of life resource use or duration of mechanical ventilation.^21,22,23,24,27,28,29,30,33^ Only 1 trial (comparing an improved end-of-life conference and bereavement leaflet vs usual care) observed a significant increase in withdrawal of life-sustaining therapy, including mechanical ventilation (27% vs 14%; *P* = .03) and vasopressors (51% vs 30%; *P* = .001).^24^ Curtis et al^23^ randomized patients and families to a communication facilitator and, while this study did not demonstrate a difference in the probability of withdrawal of life-sustaining therapy, patients in the intervention arm had a 9-day reduction in time to withdrawal compared with the control arm (7.2 vs 16.5 days; *P* = .001). Two trials by Schneiderman and colleagues^28,29^ comparing a preemptive ethics consultation with standard care showed a significant reduction in duration of mechanical ventilation only among patients who died; their first trial showed a 7.7-day reduction (3.7 vs 11.4 days; *P* = .05),^28^ and their second trial showed a 1.7-day reduction (6.5 vs 8.2 days; *P* = .03).^29^ Four RCTs compared do not resuscitate documentation between the intervention and control groups: none of these RCTs showed that the interventions were associated with changes in DNR status.^21,22,28,33^

### SDM and Family-Related Outcomes

Associations between SDM and family-centered outcomes were inconsistent. All 13 trials reported outcomes centered on a family member or SDM. Six trials assessed anxiety and depression at follow-up in family members.^23,24,25,26,30,32^ Four of these trials also assessed PTSD,^23,24,26,30^ with 1 trial of an early palliative care consultation showing an increase in PTSD.^30^ Most of these trials were amenable to meta-analysis, which demonstrated no significant association with depression (ratio of means, −0.11; 95% CI, −0.29 to 0.08; *P* = .26), anxiety (ratio of means, −0.08; 95% CI, −0.25 to 0.08; *P* = .31), or PTSD (ratio of means: −0.04; 95% CI, −0.21 to 0.13; *P* = .65) (eFigure 1 in the Supplement). Only the RCT by Lautrette et al^24^ of an improved end-of-life conference and bereavement leaflet observed a consistent reduction of anxiety and depression (median Hospital Anxiety and Depression Scale score of 11 in the intervention group vs 17 in the control group; *P* = .04), and PTSD (median Impact of Event Scale of 27 vs 39; *P* = .02) at a 3-month follow-up. Statistical heterogeneity, as determined by the *I*^2^ measure, was estimated to be moderate for depression (*I*^2^ = 69%) and anxiety (*I*^2^ = 62%) and substantial for PTSD (*I*^2^ = 77%).

Two trials assessing a media-based intervention (information leaflet^32^ and educational video^33^) evaluated comprehension in family members and showed improved understanding but no change in patient- or family-centered outcomes. There was no difference between the intervention and the control groups in 6 trials assessing satisfaction with care or perceived quality of care.^21,22,27,30,31,32^ Baseline satisfaction with care before randomization was not measured in these trials.

### Resource Use

Six trials reported different outcomes centered on resource use (cost or resource use or involvement of medical teams).^21,22,23,26,27,31^ Only the communication facilitator-led intervention used by Curtis et al^23^ led to a significant reduction in costs ($75 850 control vs $51 060 intervention; *P* = .04), which was more pronounced among patients who died ($98 220 control vs $22 690 intervention; *P* = .03). In a follow-up economic feasibility analysis of the trial, both short-term (direct variable) and long-term total health care costs were reduced.^34^ Although cost-effectiveness analyses were not provided, White et al^26^ estimated the cost of their multicomponent family support team at $170 per patient.

## Discussion

To our knowledge, this is the first systematic review and meta-analysis to focus on interventions aimed at the SDM in the ICU and their association with the course of the patient and family, as well as the first to meta-analyze major patient- and family-centered outcomes. In aggregate, these protocolized interventions were associated with a reduction in ICU LOS by approximately 2 days among patients who died but had no association with overall mortality. Taken together, these findings suggest that such interventions may be associated with beneficial patient-centered outcomes, such as reduced duration of life support among those who appeared unlikely to survive, while not influencing overall survival. However, data on withdrawal of life-sustaining therapy was inconsistently available to confirm this as the possible explanation for the findings. These interventions may be associated with improved SDM comprehension, although their association with satisfaction with care was neutral. Although there was no difference in psychological comorbidities (ie, depression, anxiety, and PTSD) when pooled, the increase in PTSD in a trial of preemptive palliative care consultation^30^ may signal potential harm from such an intervention. In addition, although data on cost were not consistently available, a reduction in ICU LOS may be associated with lower health care costs, although the savings are likely to be substantially lower than appreciated.^35^

Interventions to support SDM decision-making were not associated with overall ICU survival for 2 reasons. First, other study outcomes are less likely to be biased by differential rates of mortality and related follow-up between the groups. Second, because prognostic opinions from ICU physicians may be erroneous, such interventions may have led to premature withdrawal of life support among patients who may have otherwise survived.^36^ Furthermore, our analysis found that interventions aimed at the SDM were associated with a 2-day reduction in ICU LOS in nonsurvivors without increasing the overall mortality rate, possibly implying that the interventions reduced potentially nonbeneficial care among patients who were unlikely to survive. This finding may reflect an overall favorable association of such standardized interventions with shared decision-making, as they may help families make difficult goals of care decisions when the prognosis is poor.

Comparable trials of interventions targeting elderly patients or patients with terminal illnesses outside the ICU have found improved patient-centered outcomes.^37^ In patients with advanced cancer, proactive end-of-life discussions between physicians, patients, and surrogates are associated with earlier hospice care, less aggressive medical therapy, lower rates of ICU admission, and mechanical ventilation and improved patient and caregiver quality of life.^38^ Similarly, providing a decision aid to surrogates of patients with advanced dementia improved their knowledge and communication and reduced decisional conflict regarding feeding options.^39^

There are several possible reasons that may explain why similar interventions targeted at patients or SDMs outside of the ICU are more consistently associated with improved patient-centered outcomes. First, surrogate decision-making and advance care planning are complex tasks, especially in acute care settings. Many patients do not discuss goals of care with their families and, even if they did, it would be implausible to expect them to have conversed in detail about the vast number of potential circumstances that could be encountered in a modern ICU. Moreover, standardizing implementation of certain interventions, for example, an ethics consultation, may not be associated with differences in patient- or SDM-centered outcomes in cases where no significant ethical or decisional conflict is evident; the findings from trials examining selective vs more routine deployment of ethics-based interventions were similar.^27,28,29^ Also, certain family members may not be comfortable acting as primary decision-maker.^40^ Furthermore, SDMs often rely on their own best interest or mutual interest when faced with decision-making.^41^ In addition, individual characteristics related to the SDM, such as coping strategies and competing responsibilities, can act as important factors; those who were faced with a similar situation in the past often have an easier time than those who are acting as SDM for the first time.^42^ Ultimately, it is possible that no single common intervention could properly target all of these concurrent processes. The families that we encounter are as unique and layered as our patients and interventions should best be tailored on an individual basis.

Following the recently published trial studying multicomponent family-support intervention by White et al,^26^ there is potential that such interventions may come into routine care among critically ill adults, particularly those at the highest risk of mortality. Our study highlights the challenges and opportunities in this field to date and helps place these findings in context. Future research into the processes of decision-making by surrogates may help improve understanding and may provide more appropriate and tailored interventions. Nonetheless, it is possible that interventions targeting the SDM in the ICU may be too far downstream in the process of surrogate decision-making. Hypothetically, higher-yield interventions could target earlier processes, such as completion of a well-informed and detailed advanced care directive in high-risk patients. In a prospective cohort study of ICU patients with acute respiratory distress syndrome and their surrogates, having prior discussions centered on advanced care planning was associated with less decisional conflict.^43^ Other interventions could educate patients and their surrogates about the implications of critical illness, the consequences of artificial life-sustaining measures, end-of-life goals of care, and the importance of communicating one’s preferences while still capable. Successful deployment of such interventions in the ICU would require interaction with the health care team for appropriate patient and SDM selection based on individual case factors to maximize effectiveness. In the ICU, physicians and health care professionals should continue to apply a shared decision-making model for treatment decisions and individualize their approach to meet the needs of the SDM.

### Limitations

This study has several limitations. First, the heterogeneity of the interventions, methods, and outcomes limits the quality of the pooled analyses. These results are most relevant among SDMs for patients already admitted to the ICU. There may be an inherent selection bias whereby such patients may have already articulated a preference for receiving intensive care or whereby physicians caring for them believe that they may have reversible acute illnesses. The extent of such preexisting dialogue was uncertain but may be factors in unmeasured heterogeneity in this review and limit generalizability of the findings outside of ICUs. Although the clinical interventions varied, all were anchored in the betterment of communication and education between family members and the health care professional. Nonetheless, given the diverse and inherently complex and multifaceted nature of the interventions, it was not possible to determine which key components were associated with favorable outcomes, rendering clinical applicability challenging. In addition, there was likely heterogeneity among family members and SDMs, which is difficult to quantify and may have been factors in the observed results. Furthermore, surrogates refusing the interventions may differ in comparison with those who were willing to accept the intervention. Regarding caregiver psychological outcomes, although there was moderate to substantial heterogeneity, we did not prespecify subgroup analyses or a meta-regression to explore factors that may explain heterogeneity; given the small number of trials contributing to each outcome, such analyses would have been underpowered.

## Conclusions

In this systematic review and meta-analysis, interventions to improve surrogate decision-making in the ICU were associated with reduced ICU LOS only among patients who died; no association with mortality was noted. Although these interventions may be associated with improvements in surrogate understanding, the outcome of family well-being and mental health is unclear. Because SDMs have considerable responsibility for shared decision-making for many ICU patients, a better understanding of the complex processes related to surrogate decision-making is needed so that future trials of interventions aimed at the SDM lead to improved outcomes.

## Notes

Supplement. **eTable 1.** Medline (Ovid) Search Strategy **eTable 2.** Risk of Bias Assessment Using the Cochrane Risk of Bias Tool for Randomized Controlled Trials **eTable 3.** Summary of Individual Trial Results **eFigure 1.** Forest Plots for Psychological Comorbidities in Family Members, Including Depression, Anxiety, and PTSD **eFigure 2.** Funnel Plot for All-Cause Mortality **eReferences.**
