# Supplementary material for: Association of Surrogate Decision-making Interventions for Critically Ill Adults With Patient, Family, and Resource Use Outcomes: A Systematic Review and Meta-analysis
Source: JAMA Netw Open. 2019 Jul 19;2(7):e197229. doi: 10.1001/jamanetworkopen.2019.7229 (PMC6646989; doi:10.1001/jamanetworkopen.2019.7229)
Supplement: Supplement. — eTable 1. Medline (Ovid) Search Strategy eTable 2. Risk of Bias Assessment Using the Cochrane Risk of Bias Tool for Randomized Controlled Trials eTable 3. Summary of Individual Trial Results eFigure 1. Forest Plots for Psychological Comorbidities in Family Members, Including Depression, Anxiety, and PTSD eFigure 2. Funnel Plot for All-Cause Mortality eReferences. [file jamanetwopen-2-e197229-s001.pdf]

Supplementary Online Content

Bibas L, Peretz-Larochelle M, Adhikari NK, et al. Association of surrogate decision-making interventions for critically ill adults with patient, family, and resource use outcomes: a systematic review and meta-analysis. *JAMA Netw Open*. 2019;2(7):e197229. doi:10.1001/jamanetworkopen.2019.7229

eTable 1. Medline (Ovid) Search Strategy

eTable 2. Risk of Bias Assessment Using the Cochrane Risk of Bias Tool for Randomized Controlled Trials

eTable 3. Summary of Individual Trial Results

eFigure 1. Forest Plots for Psychological Comorbidities in Family Members, Including Depression, Anxiety, and PTSD

eFigure 2. Funnel Plot for All-Cause Mortality

eReferences.

This supplementary material has been provided by the authors to give readers additional information about their work.

© 2019 Bibas L et al. *JAMA Network Open.*

eTable1. Medline (Ovid) search strategy

#	Searches
1	exp Advance Directives/ and Decision Making/
2	Advance Care Planning/
3	Living Wills/
4	Proxy/
5	Third-Party Consent/
6	Presumed Consent/
7	Patient Preference/ and Decision Making/
8	exp Family/ and Decision Making/
9	Critically ill/ and Decision Making/
10	Terminally Ill/ and Decision Making/
11	Caregivers/ and Decision Making/
12	Legal Guardians/ and Decision Making/
13	Moral Obligation/ and Decision Making/
14	Personal Satisfaction/ and Decision Making/
15	Advance? Care Planning.mp.
16	Advance? Care Directive?.mp.
17	(alternate? adj3 (decision-mak* or consent*)).mp.
18	(alternative?? adj3 (decision-mak* or consent*)).mp.
19	((carer? or caregiver? or care-giver?) adj3 (decision-mak* or consent*)).mp.
20	((family or families) adj3 (decision-mak* or consent*)).mp.
21	(guardian* adj3 (decision-mak* or consent*)).mp.
22	((proxy or proxies) adj3 (decision-mak* or consent*)).mp.
23	(relative? adj3 (decision-mak* or consent*)).mp.
24	(spous??? adj3 (decision-mak* or consent*)).mp.
25	(substitute? adj3 (decision-mak* or consent*)).mp.
26	(surrogate? adj3 (decision-mak* or consent*)).mp.
27	(third party?? adj2 (decision-mak* or consent*)).mp.
28	((family or families or carer? or caregiver? or care-giver? or guardian* or proxy or proxies or representative? or spous???) adj3 (share or shares or shared or sharing) adj2 (decision-mak* or consent?)).mp.
29	(consent adj2 board?).mp.
30	decisional surrogate?.mp.
31	(decision-making adj2 representative?).mp.
32	(health* adj2 (proxy or proxies)).mp.
33	("living will" or "living wills").mp.
34	substitute health-care decision*.mp.
35	substitute healthcare decision*.mp.

© 2019 Bibas L et al. *JAMA Network Open.*

36	(surrogacy and (decision* adj3 authorit*)).mp.
37	surrogate consenter?.mp.
38	(surrogate and (decision* adj3 authorit*)).mp.
39	third party decision*.mp.
40	"power? of attorney".mp.
41	(patient?? adj1 agent?).mp.
42	(patient? adj2 (proxy or proxies)).mp.
43	(patient?? adj2 (surrogate? or surrogacy)).mp.
44	attorney for personal.mp.
45	attorney-in-fact.mp.
46	public guardian?.mp.
47	public trustee?.mp.
48	**or/1-47 [Substitute Decision Makers & related terms]**
49	exp randomized controlled trial/ [MeSH]
50	exp randomized controlled trials as topic/ [MeSH]
51	randomized controlled trial.pt.
52	(random* adj10 (study or studies or trial?)).mp,kw.
53	Random Allocation/
54	doubleblind*.mp.
55	Double-Blind Method/
56	((single or double or triple or treble) adj6 (blind* or mask* or conceal* or allocat*)).mp,kw.
57	exp Meta-Analysis/
58	meta-analysis as topic/
59	meta-analysis.pt.
60	(meta-anal* or metanal* or metaanal*).mp,kw.
61	metareview*.mp,kw.
62	meta-review*.mp,kw.
63	(systematic adj4 (review or reviews or overview or overviews)).mp,kw.
64	(umbrella adj3 review?).mp,kw.
65	**or/49-64 [RCTs or SRs or MAs]**
66	**48 and 65 [Substitute Decision Makers + RCTs /SRs/MAs]**
67	limit 66 to (english or french)
68	remove duplicates from 67

© 2019 Bibas L et al. *JAMA Network Open.*

eTable 2. Risk of Bias Assessment Using the Cochrane Risk of Bias Tool for Randomized Controlled Trials

First Author & year	Random Sequence Generation	Allocation Concealment	Blinding of participants and researchers	Blinding of outcome assessment	Incomplete outcome data	Selective reporting
Andereck[1] 2014	Low	Unclear	High	Unclear	Low	High
Azoulay[2] 2002	Low	Low	High	Low	Low	Low
Carson[3] 2016	Low	Unclear	High	Low	Unclear	Low
Cheung[4] 2010	Low	Low	High	Unclear	High	Low
Connors[5] 1995	High	Unclear	High	Low	Low	Low
Curtis[6] 2011	Low	Unclear	High	Unclear	Low	Low
Curtis[7] 2016	Unclear	Low	High	Unclear	Low	Low
Lautrette[8] 2007	Low	Low	High	Low	Low	Low
Schneiderman[9] 2000	Unclear	Unclear	High	Unclear	Low	Low
Schneiderman[10] 2003	Low	Low	High	Low	Low	Low
Torke[11] 2016	Low	Low	High	Unclear	High	Low
White[12] 2018	Low	Unclear	High	Low	Low	Low
Wilson[13] 2015	Low	Low	High	Low	Low	Low

© 2019 Bibas L et al. *JAMA Network Open.*

eTable 3: Summary of individual trial results

Category	First Author (year)	Patient-Related	SDM-Related	Resource-Utilization
Healthcare professional	Connors (1995)[5]	Hospital/6m mortality: ND<br>Composite of ICU LOS, MV or comatose: ND<br>Satisfaction with care: ND<br>Documentation of DNR: ND<br>Discussion about CPR: ND	Satisfaction with care: ND<br>Discussion about CPR: ND	Hospital resources: ND
	Curtis (2011)[6]	ICU LOS: ND<br>DNR orders: ND<br>WLST: ND	Satisfaction with care: ND<br>Quality of death: ND	Palliative care team consultation: ND
	Curtis (2016)[7]	ICU/Hospital mortality: ND<br>ICU LOS: ND but shorter amongst decedents (7.7d vs 28.5d)<br>Hospital LOS: Reduced (24.1d v 32.6d)<br>WLST among deceased: ND<br>Time to WLST among deceased: Reduced (7.2d v 16.5d)	Depression: Decreased symptoms at 6 months (ND at 3 months)<br>Anxiety and PTSD at 3 and 6 months: ND	ICU and hospital costs: Decreased, especially among decedents
	Lautrette (2007)[8]	Mortality: ND<br>ICU LOS: ND<br>WLST: Reduction in MV (27% v 14%), Vasopressors (51% v 30%) but ND in dialysis	Depression, anxiety, PTSD: Reduction at 3 months<br>Conflict between staff and family: ND	NA
	Torke (2016)[11]	NA	Depression, anxiety, PTSD at 6-8 weeks: ND<br>Decision conflict/regret: ND	NA
	White (2018)[12]	In-hospital mortality: Increased<br>Six-month mortality: ND<br>Reduced ICU LOS 6.7d v 7.4d<br>Reduced hospital LOS: 10.4d v 13.5d<br>Independent living: ND	Depression, anxiety, PTSD at 6 months: ND<br>Improved quality of communication with intervention	Mean cost of intervention: $170 per patient
Ethics	Andereck (2014)[1]	Mortality: ND<br>ICU and hospital LOS: ND<br>Length of MV: ND<br>Artificial nutrition: ND	Quality of care: ND	Cost of hospital stay: ND

© 2019 Bibas L et al. *JAMA Network Open.*

	Schneiderman (2000)[9]	Mortality: ND<br>CPR/MV/DNR order: ND<br>ICU LOS in all: ND<br>Amongst decedents, reduced ICU LOS (4.2d v 13.2d), MV (3.7d v 11.4d), artificial nutrition (4.1d v 12.0d)	Intervention was regarded favourably	NA
	Schneiderman (2003)[10]	Mortality: ND<br>ICU/Hospital LOS/MV in all: ND<br>Amongst decedents, reduction in hospital LOS (-2.95 d), ICU LOS (-1.44d), MV (-1.7d)	Intervention was deemed helpful	NA
Palliative care	Carson (2016)[3]	Hospital and 90d mortality: ND<br>ICU/Hospital LOS: ND<br>Length of MV: ND	PTSD at 3 months: Increased<br>Anxiety/Depression at 3 months: ND<br>Satisfaction: ND<br>Quality of communication: ND<br>Discussion of patient preferences: ND	NA
	Cheung (2010)[4]	Mortality: ND<br>ICU/Hospital LOS: ND	Satisfaction: ND	Number of medical teams involved: ND
Media	Azoulay (2002)[2]	Mortality: ND<br>ICU LOS: ND	Comprehension: Improved<br>Satisfaction: ND<br>Anxiety/Depression at 3-5 days: ND	NA
	Wilson (2015)[13]	CPR knowledge: improved<br>Resuscitation preferences: ND<br>Documented change in code status: ND<br>Utilization of CPR or MV: ND	Comprehension of CPR and other options: improved	NA

Abbreviations: CPR = cardio-pulmonary resuscitation; d = days; LOS = length of stay; MV = mechanical ventilation; ICU = intensive care unit; NA = not assessed; ND = no difference; WLST = withdrawal of life-sustaining therapies.

© 2019 Bibas L et al. *JAMA Network Open.*

eFigure 1. Forest plots for psychological comorbidities in family members, including (A) depression, (B) anxiety, and (C) PTSD.

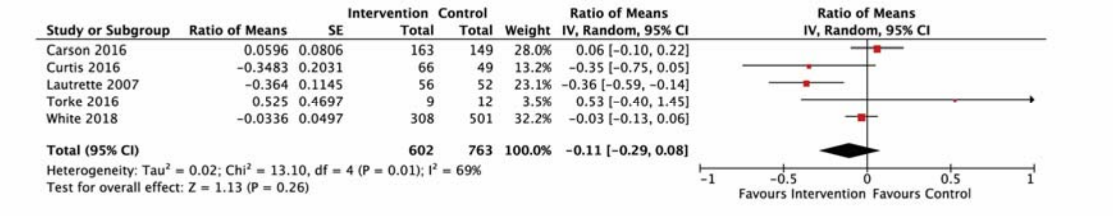

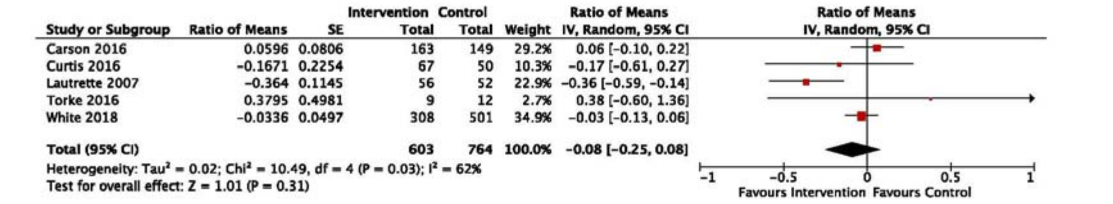

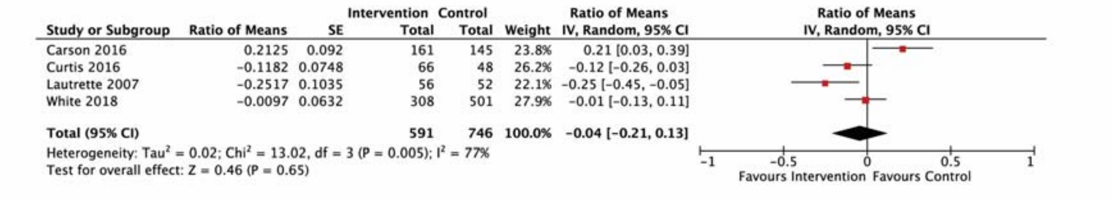

© 2019 Bibas L et al. *JAMA Network Open.*

eFigure 2. Funnel plot for all-cause mortality.

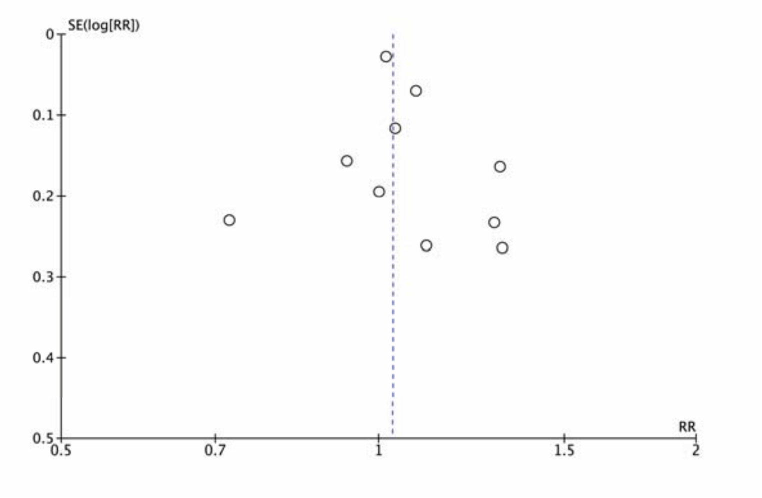

© 2019 Bibas L et al. *JAMA Network Open.*

eReferences.

1. Andereck WS, McGaughey JW, Schneiderman LJ, Jonsen AR. Seeking to reduce nonbeneficial treatment in the ICU: an exploratory trial of proactive ethics intervention. Crit Care Med. 2014;42(4):824-830.
2. Azoulay E, Pochard F, Chevret S, et al. Impact of a family information leaflet on effectiveness of information provided to family members of intensive care unit patients: a multicenter, prospective, randomized, controlled trial. Am J Respir Crit Care Med. 2002;165(4):438-442.
3. Carson SS, Cox CE, Wallenstein S, et al. Effect of palliative care–led meetings for families of patients with chronic critical illness: a randomized clinical trial. JAMA. 2016;316(1):51-62
4. Cheung W, Aggarwal G, Fugaccia E, et al. Palliative care teams in the intensive care unit: a randomised, controlled, feasibility study. Crit Care Resusc. 2010;12(1):28-35
5. Connors AF, Dawson NV, Desbiens NA, et al; The SUPPORT Principal Investigators. A controlled trial to improve care for seriously ill hospitalized patients: the Study to Understand Prognoses and Preferences for Outcomes and Risks of Treatments (SUPPORT). JAMA. 1995;274(20):1591-1598
6. Curtis JR, Nielsen EL, Treece PD, et al. Effect of a quality-improvement intervention on end-of-life care in the intensive care unit: a randomized trial. Am J Respir Crit Care Med. 2011;183(3):348-355
7. Curtis JR, Treece PD, Nielsen EL, et al. Randomized trial of communication facilitators to reduce family distress and intensity of end-of-life care. Am J Respir Crit Care Med. 2016;193(2):154-162
8. Lautrette A, Darmon M, Megarbane B, et al. A communication strategy and brochure for relatives of patients dying in the ICU. N Engl J Med. 2007;356(5):469-478
9. Schneiderman LJ, Gilmer T, Teetzel HD. Impact of ethics consultations in the intensive care setting: a randomized, controlled trial. Crit Care Med. 2000;28(12):3920-3924
10. Schneiderman LJ, Gilmer T, Teetzel HD, et al. Effect of ethics consultations on nonbeneficial life-sustaining treatments in the intensive care setting: a randomized controlled trial. JAMA. 2003;290(9):1166-1172
11. Torke AM, Wocial LD, Johns SA, et al. The family navigator: a pilot intervention to support intensive care unit family surrogates. Am J Crit Care. 2016;25(6):498-507
12. White DB, Angus DC, Shields A-M, et al. A randomized trial of a family-support intervention in intensive care units. N Eng J Med. 2018;378(25):2365-2375
13. Wilson ME, Krupa A, Hinds RF, et al. A video to improve patient and surrogate understanding of cardiopulmonary resuscitation choices in the ICU: a randomized controlled trial. Crit Care Med. 2015;43(3):621-629

© 2019 Bibas L et al. *JAMA Network Open.*
